# Supplementary material for: Characterizing meiotic chromosomes' structure and pairing using a designer sequence optimized for Hi‐C
Source: Mol Syst Biol. 2018 Jul 16;14(7):e8293. doi: 10.15252/msb.20188293 (PMC6047084; doi:10.15252/msb.20188293)
Supplement: Supplementary file 1 — Expanded View Figures PDF [file MSB-14-e8293-s001.pdf]

Expanded View Figures

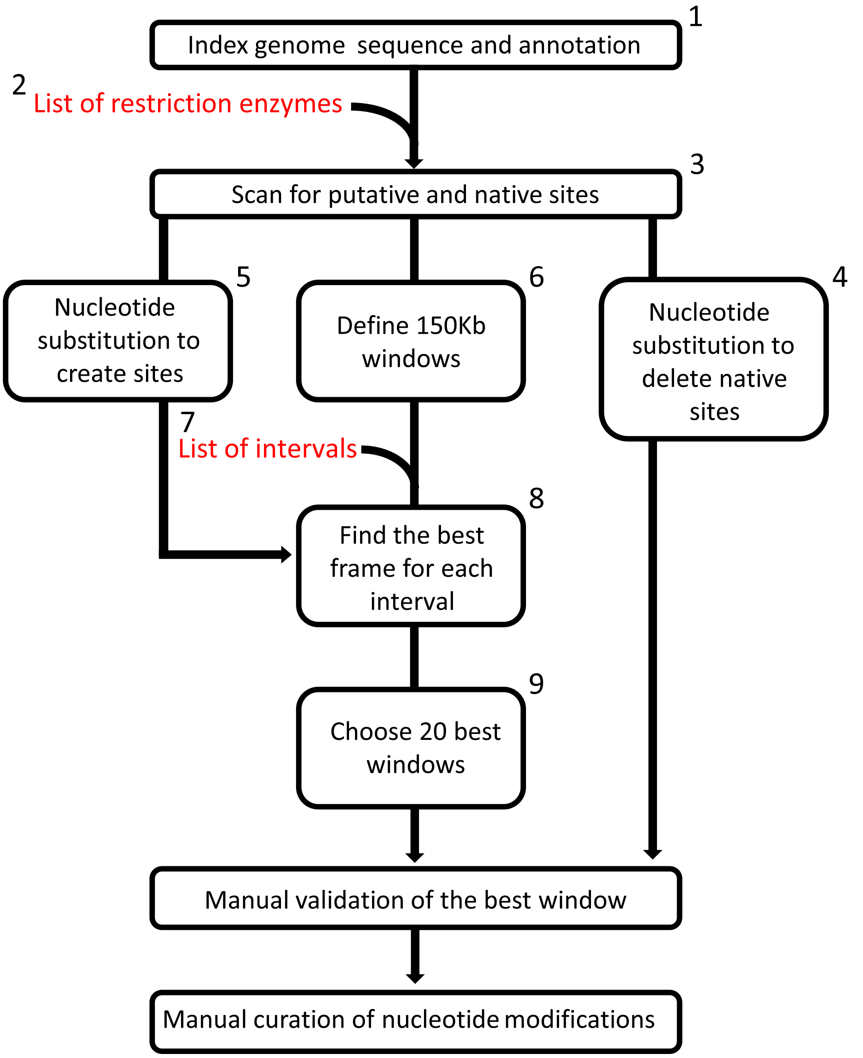

Figure EV1. Diagram of the workflow (related to Fig 1).

(1) Annotation (SK1 background) corresponds to CDS, ARS, telomere regions, retrotransposable elements, mating type loci, tRNA, Sn/Sno RNA, rDNA, ncRNA, intron motives, and TATA boxes. All those features but CDS and transposons were labeled as “forbidden”, preventing any nucleotide substitution in these regions.

(2) DpnII, HindIII, SacI, EcoRI, NdeI, SacII, SalI, XbaI, and XhoI.

(3) Putative restriction sites are DNA sequences differing with only one base pair from a RS recognized by a RE.

(4) The sequence modifications were allowed only in non-forbidden positions. In CDS, silent mutations were introduced. When two sites overlapped, the minimum changes needed were selected. When possible, we favored A ↔ G and C ↔ T substitutions. A validation step to test whether or not the deletion of one site creates a new site was performed after each modification, and if so, a new modification was sought for.

(5) Modifications to generate new sites were also only introduced at non-forbidden positions. Only silent mutations were introduced within coding regions.

(6) 583 × 150 kb windows with 10-kb overlaps were generated over the entire genome, excluding telomeres and 75 kb from each side of centromeres.

(7) Here, 400, 1,500, 2,000 and 6,000 bp.

(8) For each 150-kb window and each interval, the following steps were performed:
   - (i) for each enzyme, for each starting point: putative sites within the first bin of the window (0– 0+spacing).
   - (ii) find the putative sites at position *n*+1 at a distance interval ± 10% from position n until the end of window.

(9) For each window, a score is calculated as follows:
   - (i) for each interval, a score is calculated for each enzyme based on the median absolute deviation (MAD).
   - (ii) the best enzyme exhibiting the lowest score was chosen for each interval. Each spacing must have a different enzyme, so multiple combinations of enzymes were computed for each window.
   - (iii) The window score is calculated as the sum of the four chosen interval scores.

A final step of manual curation was performed to introduced PCRTags (Richardson *et al*, 2017).

 © 2018 Institut Pasteur

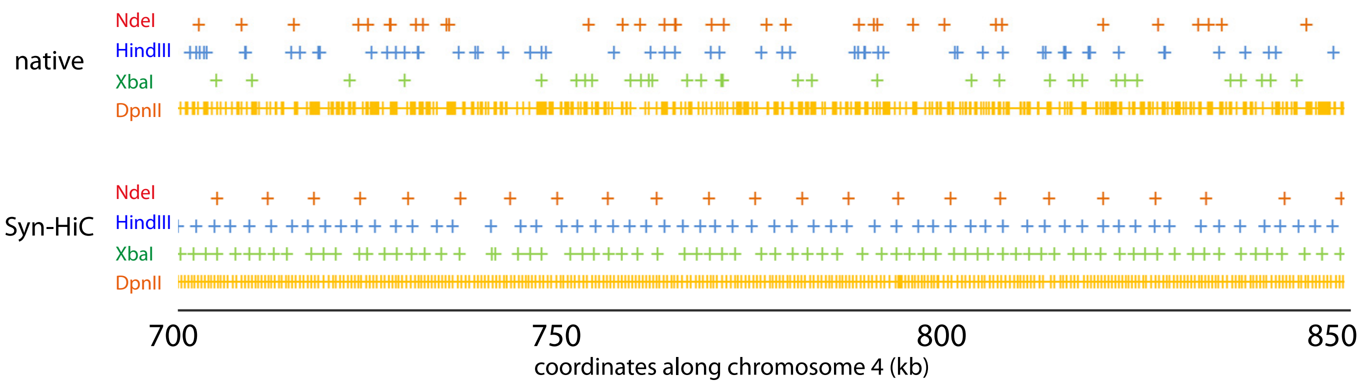

Figure EV2. Restriction pattern (related to Fig 1).

Distribution of the different restriction sites sizes in both the native sequence (top) and Syn-HiC (bottom) 150-kb redesigned sequence.

Figure EV3. Strain assembly strategy (related to Fig 1).

A Building blocks are iteratively integrated in the genome of *S. cerevisiae* through homologous recombination following transformation. An alternating auxotrophic selection and counterselection of uracil and leucine are performed to select for transformant likely to have replaced their native sequence by the redesigned one between the two extremities of the targeted region (see Muller *et al*, 2012 and Annaluru *et al*, 2014 for details).

B Full replacement of the native sequence by the synthetic one is controlled by PCR in transformants cells (SK1 background). For each transformation, PCRTags amplifying either the native or the redesigned genome confirm that the redesigned DNA was integrated over the entire region. The genome of the selected transformant is then confirmed by sequencing. The red lines delimit the region replaced for each transformation (from left to right).

C Growth curves of two independent subclones of the selected transformant and two independent subclones of the parent lineage. Each curve is computed out of eight independent cultures.

D MA plot representing the differential expression levels for all the genes between the Syn-HiC transformant and the native parental haploid strains. The ORFs within the Syn-HiC redesigned regions whose expression levels are significantly modified are indicated on the graph. Green dots: genes from the Syn-HiC region. Red dots: genes for which the $\log_{10}(P\text{-value}) < 0.05$.

Source data are available online for this figure.

© 2018 Institut Pasteur

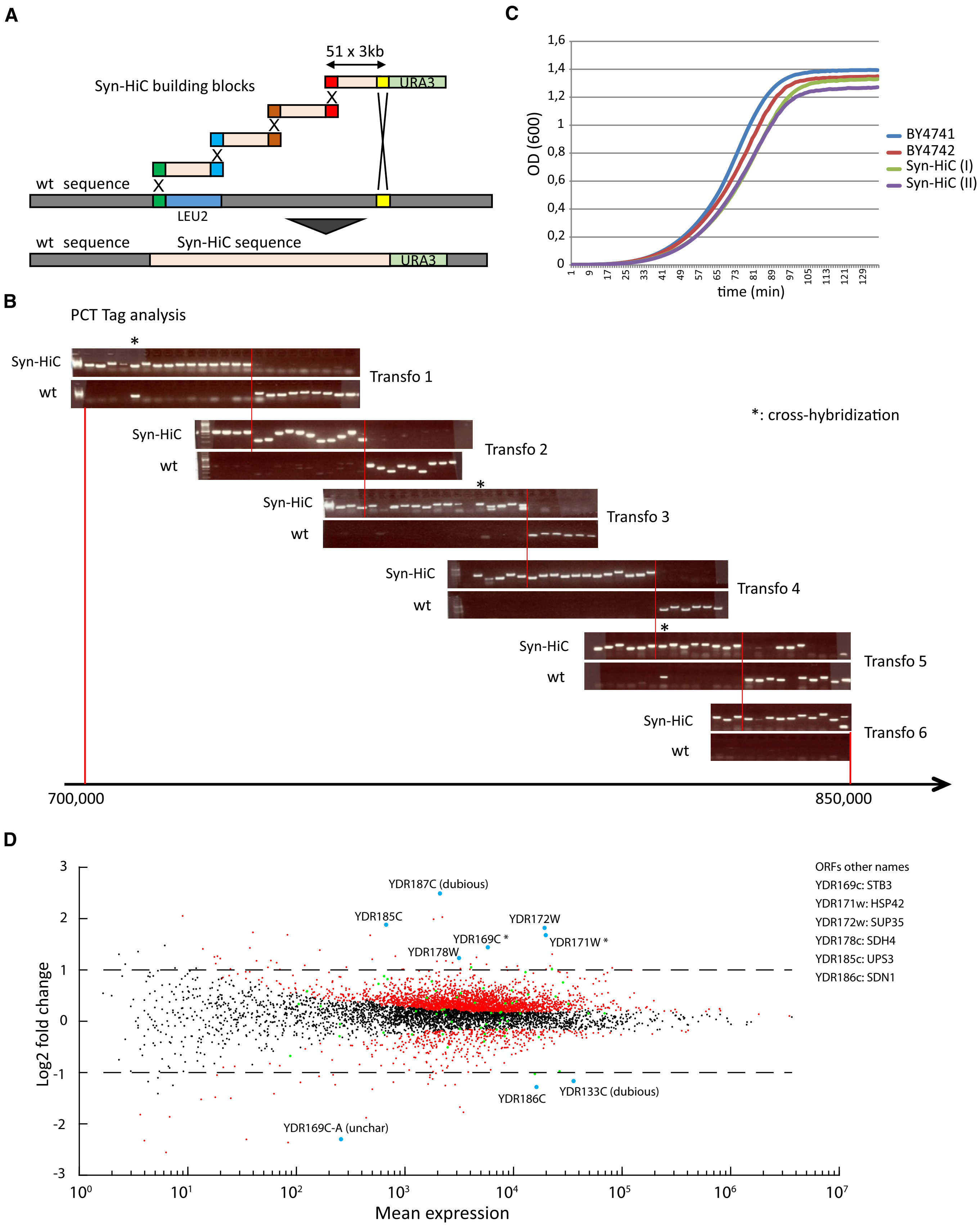

Figure EV3.

© 2018 Institut Pasteur

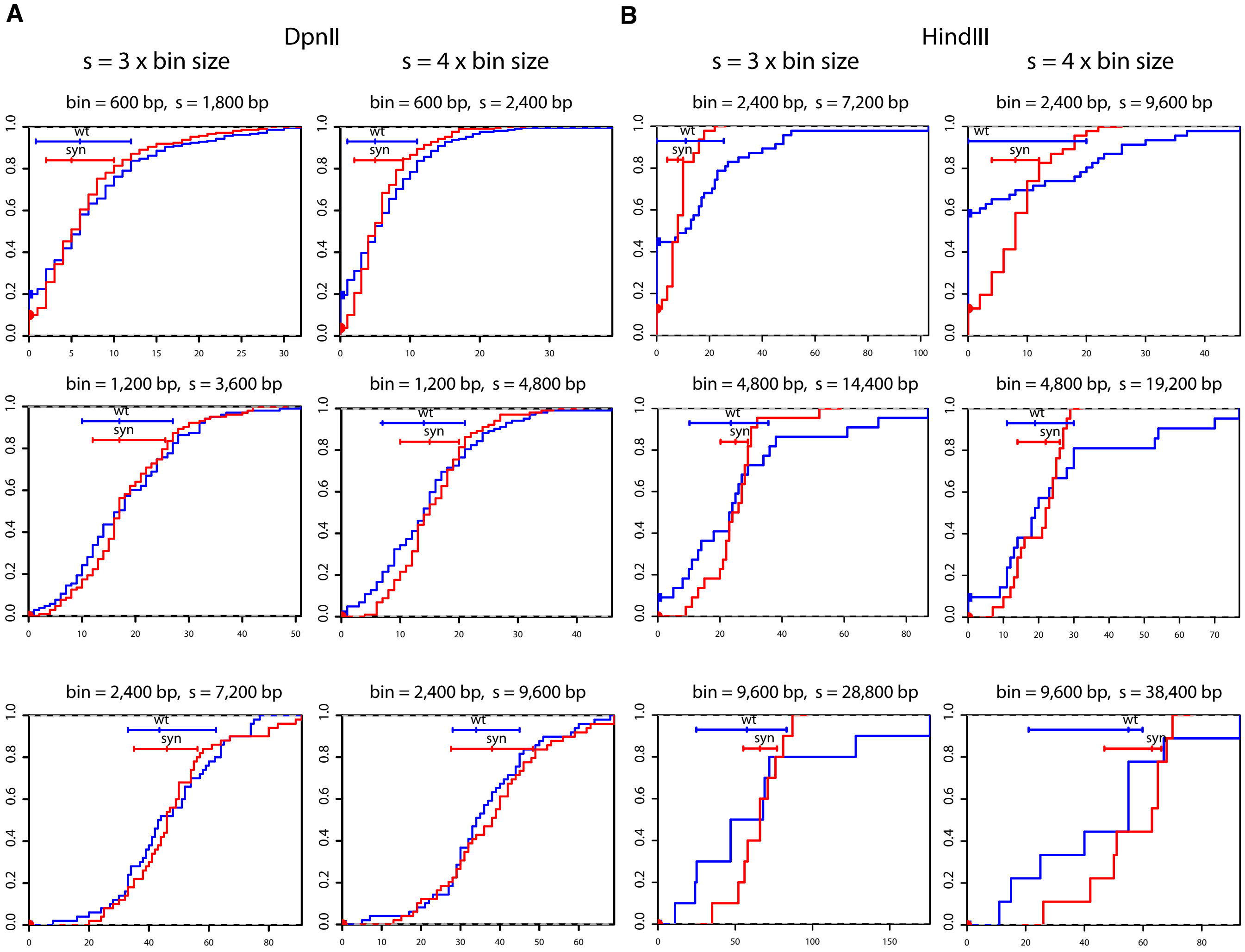

Figure EV4. Cumulative contact histograms (related to Fig 2).

A, B The cumulative histograms were computed for several values of *s*. We report the cumulative distributions of contacts as a function of *s* and bin size for DpnII (A) and HindIII (B) Hi-C contact maps. Histograms for selected values of bin sizes and distances *s* are reported in Fig 2 in the main text. Blue line: native region. Red line: synthetic region. *x*-axis: read number. *y*-axis: cumulative probability. Horizontal bars: 25th to 75th percentile. Vertical dot on the horizontal bar indicates the median. For small bin sizes (*s* = 600 bp), the distribution of contacts of the redesigned region appeared systematically narrower than for the native region, with most bins being "visible", i.e., containing at least one read. The gain in resolution somehow fades away for the frequent cutter when the bin size increases, but, interestingly, the visibility of the bins remains nevertheless systematically better. For HindIII, the gain in resolution is always considerably better in the redesigned versus the native sequence.

© 2018 Institut Pasteur

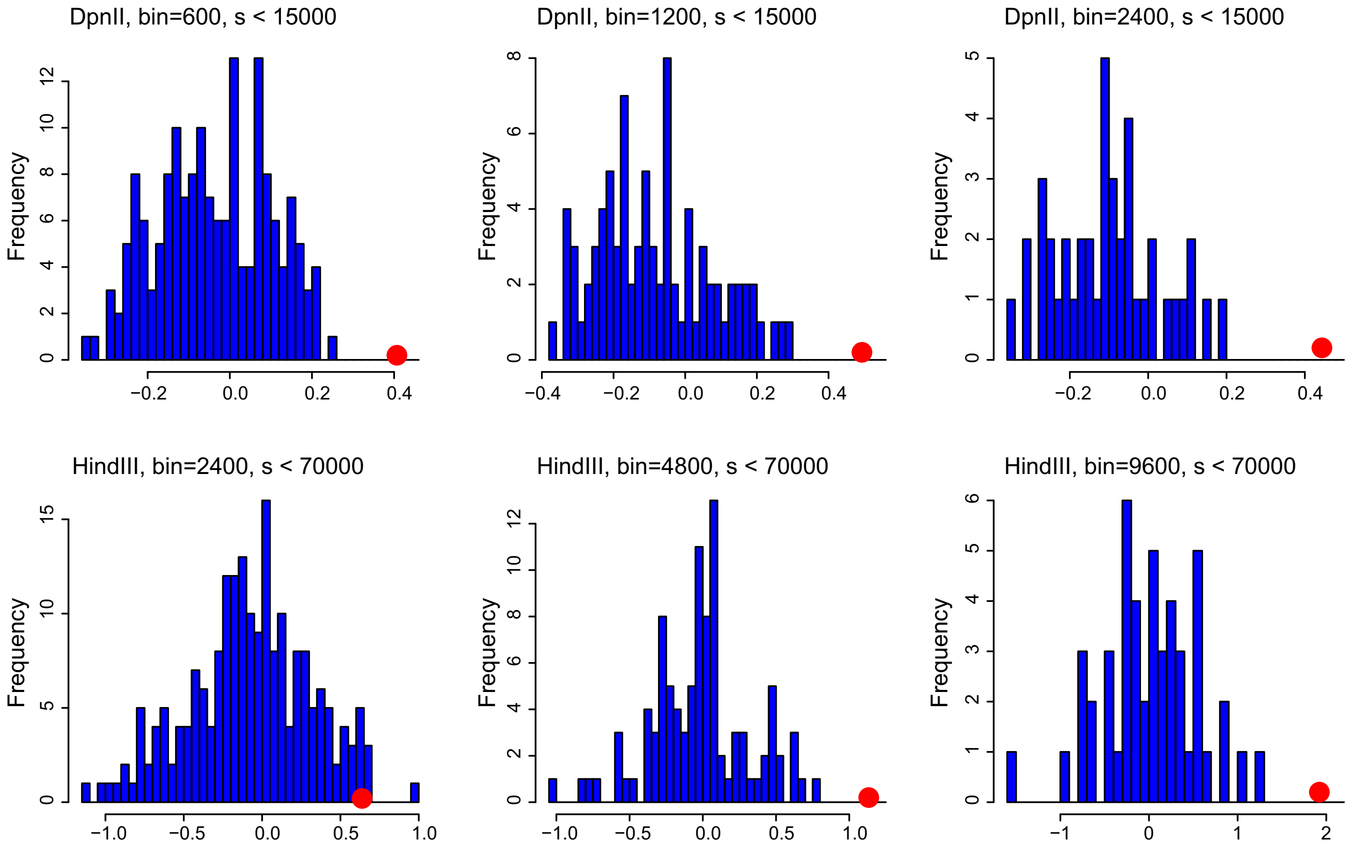

Figure EV5. Related to Fig 2.

The histogram of ratio of the CVs between Syn-HiC and wt strain, for all values of distance *s* (for *s* < 15,000 in DpnII and *s* < 70,000 in HindIII datasets) in the control regions (blue bars) compared to the mean over *s* of this value in the synthetic region (red dot). Only the synthetic region shows a quantitative improvement of statistical significance.

 © 2018 Institut Pasteur

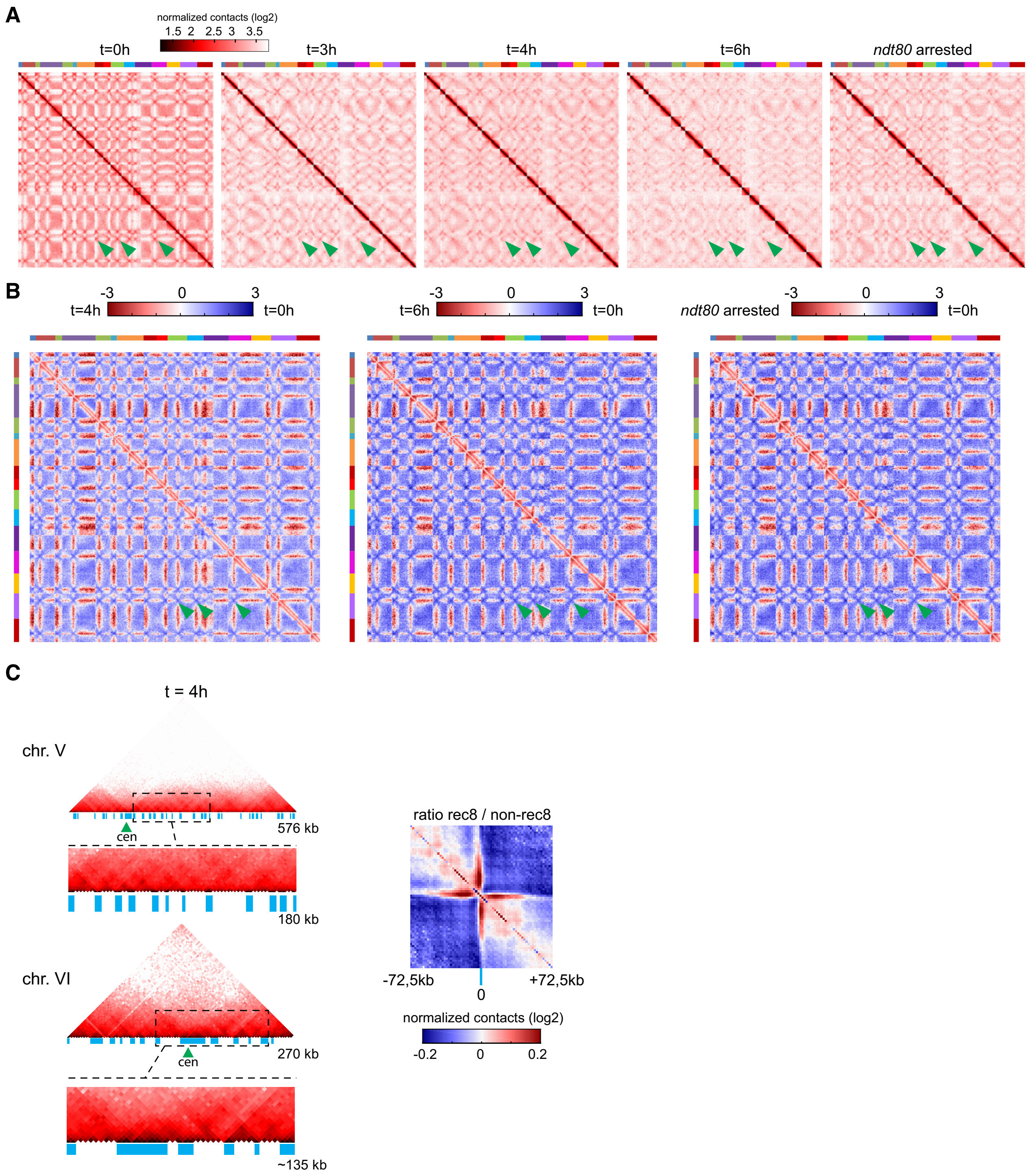

Figure EV6. Related to Figs 4 and 5.

A Normalized contact maps of synchronized populations of cells after 0, 3, 4, and 6 h in sporulation medium as well as of *ndt80Δ*-arrested pachytene cells (after 6 h in SPM). The 16 yeast chromosomes are displayed atop the maps. Green arrowheads: inter-centromeric contacts.

B Log-ratio of contact maps from (A). The blue-to-red color scale reflects the enrichment in contacts in one population with respect to the other (log2).

C Left panels: normalized contact maps of chromosomes V and VI during meiotic time course at $t = 4$ h (bin size: 5 kb). For chromosome V, a magnification of a ~180-kb region (dotted box) is displayed under the chromosome. The blue rectangles point at bins enriched in Rec8 protein. Green triangles: centromere position. For chromosome VI, a magnification of a ~135-kb region is displayed. Right panel: ratio between the cumulated normalized intra-chromosomal contacts made by 145-kb windows (2.5-kb bins) centered on Rec8-enriched bins or randomly chosen. Blue color shows a depletion of contacts in the random maps, whereas the red signal points at an enrichment in contacts in the maps centered on Rec8-enriched bins.

© 2018 Institut Pasteur
