# Supplementary figures and images for: Characterizing meiotic chromosomes' structure and pairing using a designer sequence optimized for Hi‐C
Source: Mol Syst Biol. 2018 Jul 16;14(7):e8293. doi: 10.15252/msb.20188293 (PMC6047084; doi:10.15252/msb.20188293)

## Transfo 1

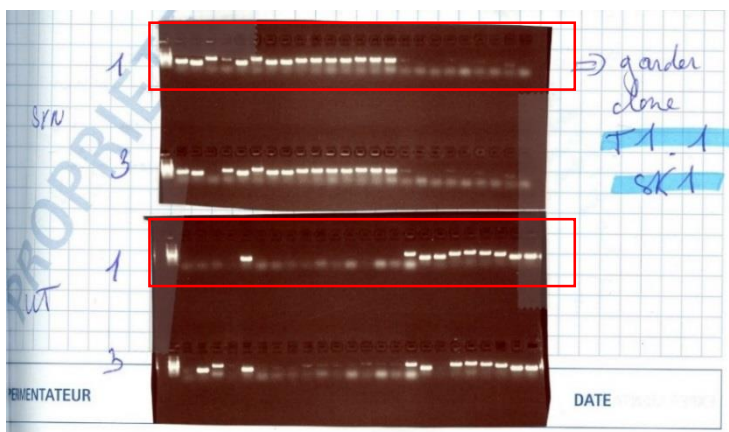

## Transfo 2

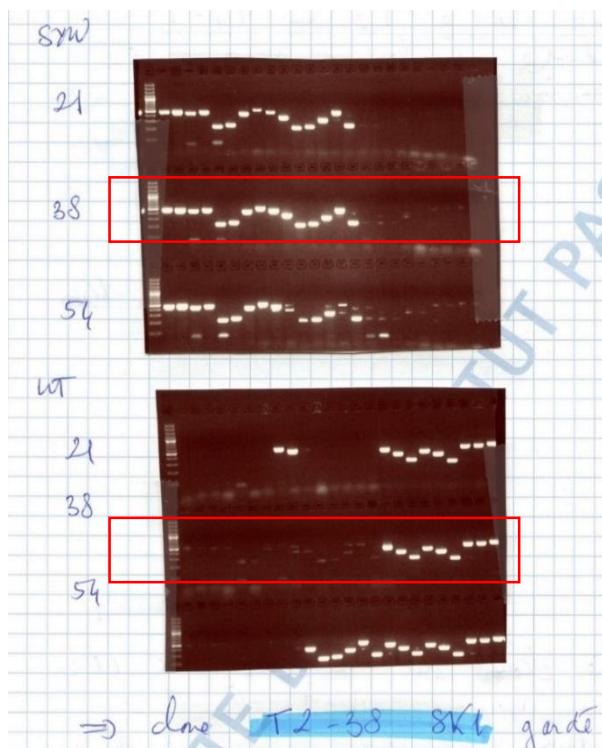

## Transfo 3

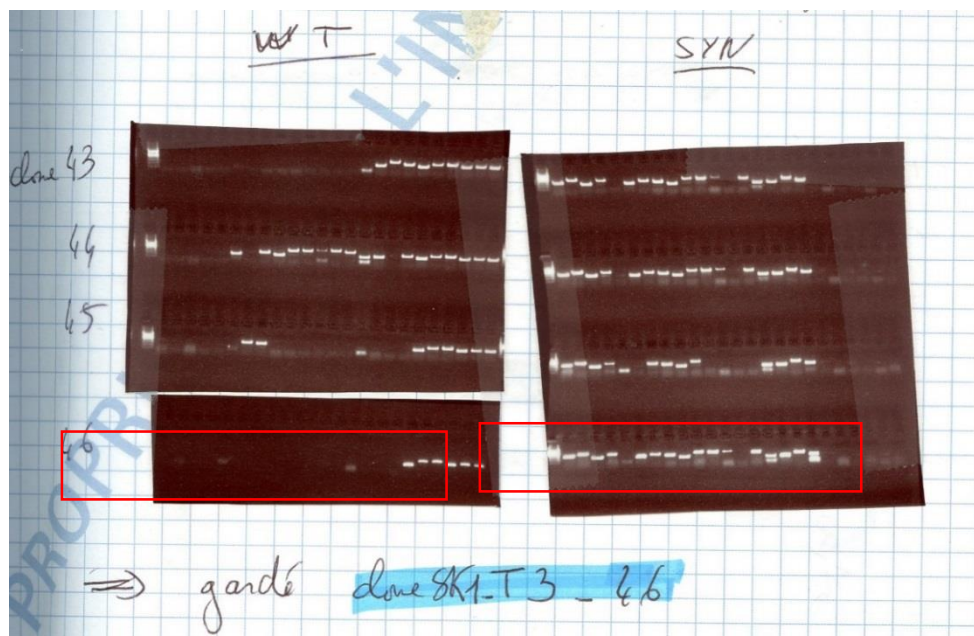

## Transfo 4

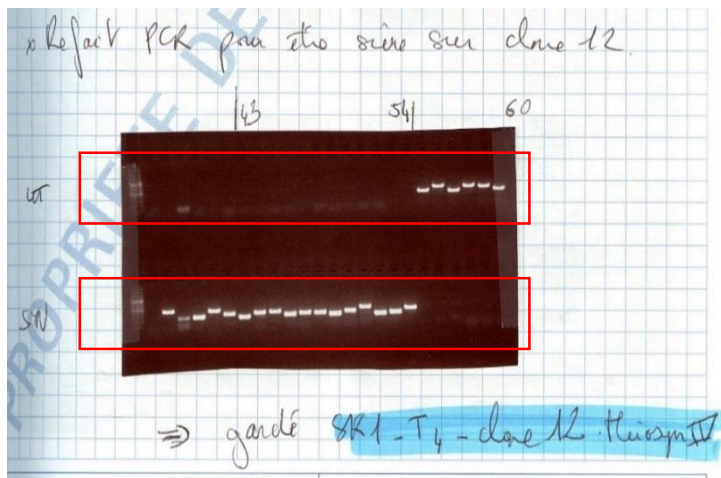

## Transfo 5

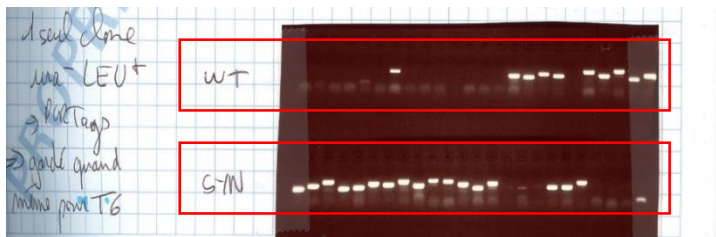

## Transfo 6

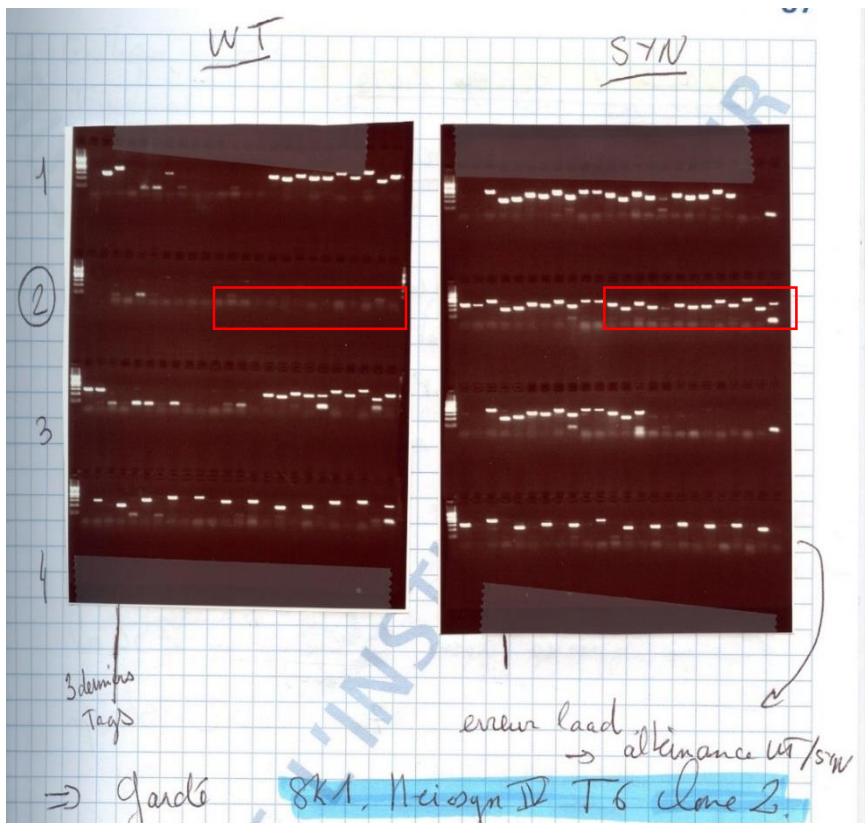

Supplement: Supplementary file 3 — Source Data for Expanded View [file MSB-14-e8293-s003.zip › source_data_Figure_EV3.pdf]
